# MoMI-G: modular multi-scale integrated genome graph browser

**DOI:** 10.1186/s12859-019-3145-2

**Published:** 2019-11-05

**Authors:** Toshiyuki T. Yokoyama, Yoshitaka Sakamoto, Masahide Seki, Yutaka Suzuki, Masahiro Kasahara

**Affiliations:** Department of Computational Biology and Medical Sciences, Graduate School of Frontier Sciences, The University of Tokyo, Chiba, Japan

**Keywords:** Genome graphs, Genome browser, Structural variant, Visualization, Variation graphs, Long-read sequencing

## Abstract

**Background:**

Genome graph is an emerging approach for representing structural variants on genomes with branches. For example, representing structural variants of cancer genomes as a genome graph is more natural than representing such genomes as differences from the linear reference genome. While more and more structural variants are being identified by long-read sequencing, many of them are difficult to visualize using existing structural variants visualization tools. To this end, visualization method for large genome graphs such as human cancer genome graphs is demanded.

**Results:**

We developed MOdular Multi-scale Integrated Genome graph browser, MoMI-G, a web-based genome graph browser that can visualize genome graphs with structural variants and supporting evidences such as read alignments, read depth, and annotations. This browser allows more intuitive recognition of large, nested, and potentially more complex structural variations. MoMI-G has view modules for different scales, which allow users to view the whole genome down to nucleotide-level alignments of long reads. Alignments spanning reference alleles and those spanning alternative alleles are shown in the same view. Users can customize the view, if they are not satisfied with the preset views. In addition, MoMI-G has Interval Card Deck, a feature for rapid manual inspection of hundreds of structural variants. Herein, we describe the utility of MoMI-G by using representative examples of large and nested structural variations found in two cell lines, LC-2/ad and CHM1.

**Conclusions:**

Users can inspect complex and large structural variations found by long-read analysis in large genomes such as human genomes more smoothly and more intuitively. In addition, users can easily filter out false positives by manually inspecting hundreds of identified structural variants with supporting long-read alignments and annotations in a short time.

**Software availability:**

MoMI-G is freely available at https://github.com/MoMI-G/MoMI-G under the MIT license.

## Background

Identifying variations and interpreting their potential impacts are critical steps toward cataloging the variations in the human genome and mechanistic understanding of genetic diseases and cancers. Hundreds of millions of single nucleotide variants have been identified in large sequencing studies [1]. With the advent of long read sequencing technologies and with the steady improvement in the preparation protocols of high-molecular-weight (HMW) DNA, larger variants including structural variations (SVs) are being identified with much higher accuracy than ever because longer reads are more uniquely aligned to the reference genome [2, 3]. Improving algorithms for calling SVs from long reads is highly demanded.

In the era of long read sequencing, a typical analysis of SVs in a species usually starts with aligning long whole-genome shotgun reads with a reference genome, after which we identify SVs as large differences between the reads and the reference genome [4, 5]. However, this approach does not work for certain regions in a genome. For example, certain sequences shared among specific populations are not present in the reference genome commonly used today [6–8]; not including these sequences in the reference genome may lead us to miss causal genetic variants of diseases. Another example is that mutations in a genomic locus with high diversity, such as the human leukocyte antigen region, are hard to identify [9]; this may also lead us to miss variants highly associated with diseases or important traits. A natural solution to this problem is to use a human pangenome that represents multiple genomes as the reference genome instead of using commonly used human reference genomes (e.g., GRCh38) which represent only a haploid sequence. A pangenome can be naturally represented by a *genome graph*, where branches are allowed and diverse sequences can be included [10, 11]. Genome graph is also referred to as *graph genome* [12, 13].

As mentioned above, genome graph is an emerging approach for extending the traditional linear genome into a genome with branches that can contain population-specific sequences and can accommodate high diversity in specific loci. Further, genome graph is proved to largely reduce reference bias that hinders accurately estimating the allele frequency [14]. However, the biggest problem with the genome graph approach is that the ecosystem around genome graphs is not yet mature compared to that for the traditional linear genomes. Genome analysis tools that support genome graphs are in urgent need, but development of genome graph analysis tools in still in the early stage. There are already several genome graph aligners [14, 15], but however, there is no genome graph browser for large genome graphs such as human genome graphs.

**Fig. 1:**
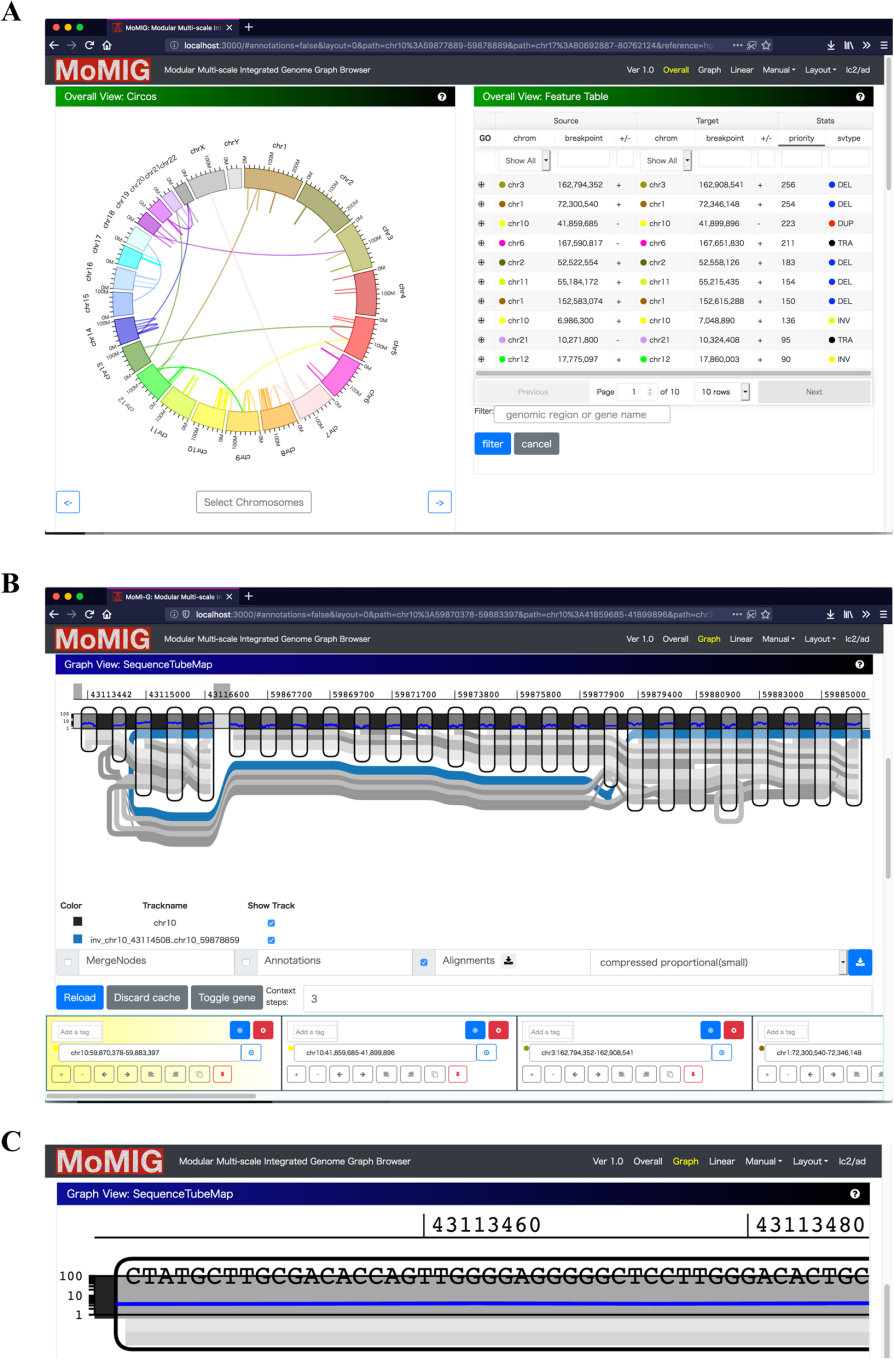
Overview of MoMI-G. A user typically selects one of the preset combinations of view modules. The user can customize the window by adding or removing view modules, if necessary. Three examples showing views of different scales are shown. Comprehensive descriptions for all modules are shown in Additional file 1: Figure S1. **a** Chromosome-scale view: Circos Plot (left) shows the distribution of SVs over all chromosomes. Arcs are chromosomes. Curves represent SVs. Feature Table (right) shows a filtered/sorted list of the SVs in an input VCF file. **b** Gene-scale view: SequenceTubeMap (top) shows the graphical view of the genomic region selected in Circos Plot, Feature Table, or Interval Card Deck. A rounded rectangle is a node that represents a piece of a genomic sequence. The thick lines spanning over nodes are paths; the horizontal thick black line with light/dark shades is a chromosome of the reference genome (a reference path); the blue line indicates an SV path that corresponds to a VCF record that represents one end of an inversion. The color of lines indicating SVs is assigned arbitrarily. Read alignment paths can be shown as gray thin lines when the read alignment information (BAM or GAM file) is provided. The inversion here is likely heterozygous. Read alignment paths are not shown in most examples shown in this paper for simplicity. Nucleotide-level alignments can be also shown on the read alignment paths if the input data contains the base-to-base alignment information (Additional file 2: Figure S2). Interval Card Deck (bottom) queues a list of genomic intervals for candidate SVs selected by using Circos Plot or Feature Table for rapidly screening hundreds of candidate intervals. **c** Nucleotide-scale view: SequenceTubeMap can show nucleotides

Here, we developed MOdular Multi-scale Integrated Genome graph browser, MoMI-G (pronounced mo-me-gee), a genome graph browser that visualizes genome graphs of up to the human-sized genomes. MoMI-G is the first-in-class genome graph browser in a sense that (1) MoMI-G can display a genome graph with long-read alignments and annotations such as genes and repetitive sequences and that (2) MoMI-G can handle genomes of up to the human-sized genomes. MoMI-G visualizes SVs using variation graphs (Fig. 1, Additional file 1: Figure S1), a variant of genome graphs [14]. Herein, we demonstrate a few examples where genome graphs and genome graph browsers provide insights into the human genome SVs. First, we describe the use cases and features of MoMI-G using the LC-2/ad human lung adenocarcinoma cell line that carries a CCDC6-RET fusion gene [16–19], and CHM1, a human hydatidiform mole cell line that originates from a single haploid [20]. MoMI-G helps us understand the entire picture of SVs, even those that are nested or large, regardless of their size. MoMI-G allows researchers to obtain novel biological knowledge by comparing a reference genome with an individual genome by using a variation graph.

Our ultimate goal is to build and visualize a large variation graph that represents a human pangenome that will provide us with insights into the human genome diversity. To include as many variants as possible in the human pangenome graph, sequencing a large number of human genomes with long read sequencing at a shallow sequencing depth [21] is a reasonable strategy in terms of the cost efficiency, although pangenome graph building tools are yet to be developed for such data. However, developing such graph building tools is hard without a visualization tool for genome graphs, because optimizing and debugging SV calling algorithms are hard unless genome graphs and aligned long reads on it are graphically visualized. Although our main contribution is the development of a genome graph browser, MoMI-G, we also developed the MoMI-G tools that convert the output of SV callers into genome graphs as a first step towards that goal. Therefore, we focus on visualizing SVs throughout this paper.

### Comparison with other SV visualization tools

Manual inspection of SVs identified using SV calling tools is important, because these tools are not yet accurate enough; human experts are required to accurately and reliably distinguish true positive SVs from false positive ones. For example, SVs identified by using reads obtained by different sequencing platforms are often not concordant [4, 5, 22], suggesting that the algorithms of SV callers need further improvement. Therefore, identified SVs need to be manually inspected using read alignments and genomic annotations [23].

Traditional genome browsers such as IGV [24–26] are popular for visualizing read alignments and annotations on a linear reference genome, but are originally not designed for long reads, and therefore the support for SVs are limited; they are not able to directly display long read alignments that align on multiple intervals of the linear reference genome. There are a few recent SV visualization tools for long reads such as svviz [27], SV-plaudit (uses samplot as a backend) [28] and Ribbon [29]. They all provide an SV-centric visualization for SVs between multiple intervals of the linear reference genome with read alignments. SV-plaudit and svviz can explicitly display SVs in multiple genomes such as trio, while MoMI-G can display multiple genomes with SVs when an external tool reconciles SVs found in multiple genomes into a single variation graph with proper paths representing SVs. svviz improves long read alignments by looking at the alignment ends of reads in multiple genomes, but MoMI-G leaves such kind of optimizations to external SV analysis tools. SV-plaudit supports showing the relationships between paired-end Illumina reads, but MoMI-G lacks the paired-end support. Ribbon provides a web interface to upload VCF files, while users have to copy input data files to a server for MoMI-G so MoMI-G requires users to have control over the web server. None of svviz, SV-plaudit (samplot), and Ribbon simultaneously display read alignments and genome annotations on more than one structurally different haplotypes. In other words, existing SV visualization tools display only one (usually local) haplotype and the associated read alignments and annotations, which makes it difficult to assess heterozygous SVs or more complex SVs often seen in cancer genomes. MoMI-G can display genome graphs with arbitrary branches and aligned reads on all branches together with annotations such as genes, repeats, the depth of Illumina reads, naturally overcoming the limitation of the existing SV visualization tools that do not support branches in a single view.

## Results

MoMI-G is a web-based genome browser developed as a single-page application implemented in TypeScript and with React. Because users need different types of views, even for the same data, MoMI-G provides three groups of view modules for the analysis of SVs at different scales, namely chromosome-scale, gene-scale, and nucleotide-scale view groups (Additional file 3: Table S1). Users can use one or more view modules in a single window.

**Fig. 2:**
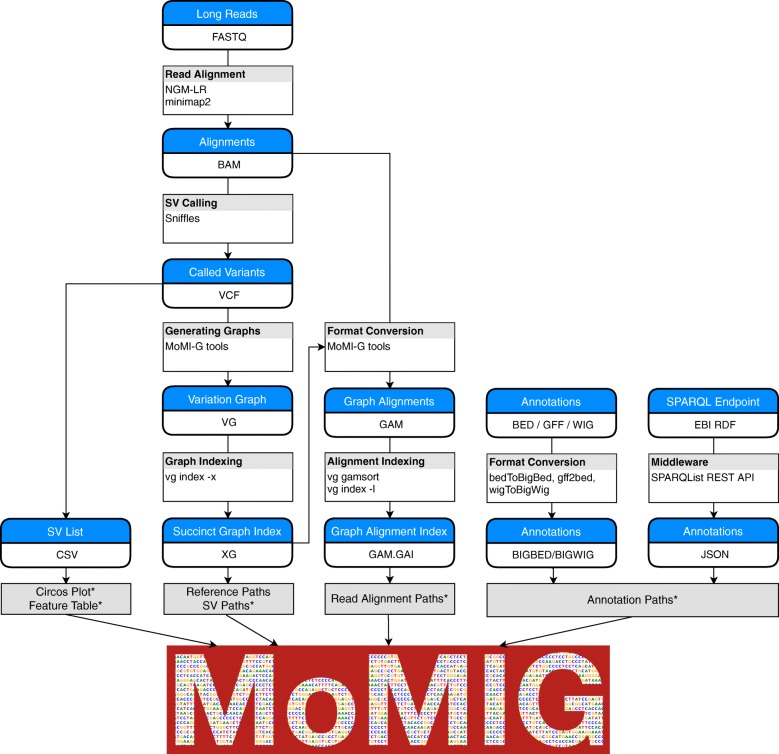
Workflow used for the LC-2/ad sample for creating the input files for MoMI-G. The workflow shown here was used for visualizing the LC2/ad sample. This is only one example of how to prepare input files for MoMI-G. Users may use other tools or other command lines. The minimum requirement is that users provide an XG file with the reference paths. Other types of information such as SV paths, read alignments, annotations are optional. An asterisk (*) indicates the input is optional

The input of MoMI-G is a variation graph, read alignment (optional), and annotations (optional). Figure 2 is a flowchart that describes a pipeline used in this paper to prepare the input data of MoMI-G from raw sequencing data and annotations. MoMI-G accepts a succinct representation of a vg variation graph, which is an XG file, as a variation graph. A script that converts a FASTA file of a reference genome and a variant call format (VCF) file into an XG file is included in the MoMI-G package, although the VCF format cannot represent some types of SVs that the XG format can represent, such as nested insertions. Read alignment data on the graph need to be represented as a graph alignment map (GAM) file in MoMI-G. One way to create a GAM file is to align reads against a reference graph using “vg map” command, i.e., remapping the reads against the reference genome augmented by the identified SVs. Another way is to convert a binary alignment map (BAM) file into a GAM file, which is a recommended way when we wish to inspect the SVs identified by an SV caller. We show three examples from two samples to demonstrate the utility of MoMI-G. One of the examples is a large inversion, and the other is nested SVs that are difficult to visualize using existing tools. We also show nested SVs from the CHM1 dataset [20]. For all the examples, we used the Amazon EC2 instance type t2.large with 8 GB of memory and 2.4 GHz Intel Xeon processor as the MoMI-G server; the server requirement for this tool is minimal. MoMI-G supports common browsers, including Chrome, Safari, and Firefox.

### Data model used in MoMI-G and MoMI-G tools

To our knowledge, no publicly available SV visualization tools are available for large and nested SVs with alignments of long reads. Thus, we aimed to develop a genome graph browser at the earliest so that users can obtain new biological knowledge from real data. We used an existing library, SequenceTubeMap [30], for visualizing a variation subgraph, rather than developing our own library from scratch.

SequenceTubeMap is a JavaScript library that visualizes multiple related sequences such as haplotypes. A variation graph used in SequenceTubeMap is a set of nodes and paths, where a node represents part of a DNA sequence, and a path represents (part of) a haplotype. Edges are implicitly represented by adjacent nodes in paths.

MoMI-G accepts variation graphs in which SVs are represented by paths so that SequenceTubeMap can visualize them. We classified paths as four types; reference paths, SV paths, read alignment paths, and annotation paths because paths can represent various kind of sequences embedded in the graph. Reference paths represent chromosomes in the reference genome. SV paths represent SVs as follows. A deletion is represented by a path that skips over a sequence that other paths pass through. Similarly, an insertion is represented by a path that passes through an extra sequence that other paths do not visit; an inversion is represented by a path where part of the sequence in other paths is reversed; and, a duplication is represented by a path that passes through the same sequence twice or more. Read alignment paths represent read alignment when the read alignment information (BAM or GAM file) is provided. Annotation paths represent annotations such as genes or repeats. These four paths are displayed different appearances on SequenceTubeMap.

The MoMI-G package includes a set of scripts (MoMI-G tools) that convert a VCF file into the variation graph format. We used MoMI-G tools for generating the input variation graphs; alternatively, users can generate variation graphs on their own. See the method section and Additional file 4: Figure S3 for details of the MoMI-G tools. Briefly, MoMI-G tools convert a VCF record into an SV path in the output variation graph. A deletion is converted into an SV path that starts, at most, 1 Mbp before one breakend of the deletion, traverses to the breakend, jumps to the other breakend, and proceeds for a certain length (< 1 Mbp). Note that the sequences flanking the deletion are added to indicate the edge representing the deletion because edges are implicitly represented in SequenceTubeMap. Insertions, inversions, and duplications are similarly represented by SV paths with flanking sequences.

## Visualization Examples

### Revealing a large SV: a large inversion and a subsequent short deletion

Using MoMI-G, we show an example of a complex SV that involves two SVs, identified by Sniffles, each of which connects two different points on a chromosome. This complex SV can be considered a large inversion and two deletions flanking the reference genome. Previous studies involving the use of whole genome sequencing or RNA-seq with the Illumina HiSeq or Nanopore MinION identified the CCDC6-RET fusion gene in LC-2/ad [17–19, 31]. However, those studies focused only on the region around the CCDC6-RET fusion point, and the entire picture, including the other end of the inversion, was unclear. To address this issue, we explored the wider region around CCDC6-RET with MoMI-G.

First, we sequenced the genome of LC-2/ad with Oxford Nanopore MinION R9.5 pore chemistry and merged reads with those from a previous study (accession No. DRX143541-DRX143544) [31]. We generated 3.5 M reads to 12.8× coverage in total and then aligned them with GRCh38. The average length of the aligned reads was 16 kb (Additional file 5: Table S2). We detected 11,316 SVs in the VCF format, including the previously known CCDC6-RET fusion gene, on the nuclear DNA of LC-2/ad cell line (Additional file 6: Table S3). See the methods section for details.

**Fig. 3:**
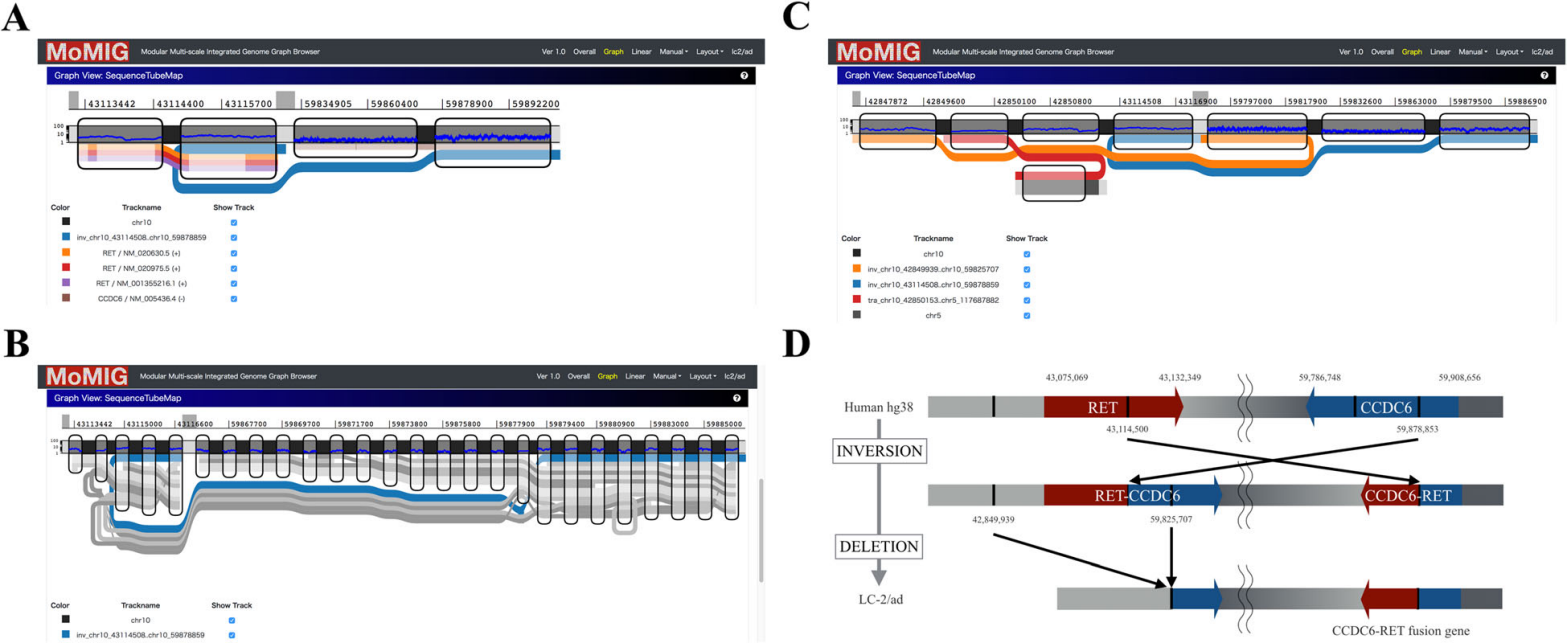
Example of CCDC6-RET. **a** CCDC6-RET shown in MoMI-G (compressed view). The thickest black line is chromosome 10 (reference genome). Note that the two distinct intervals of chromosome 10 are shown, which correspond to the RET (left interval) and CCDC6 (right interval) genes. The blue line represents an inversion identified by Sniffles, showing the CCDC6-RET fusion event. The other lines are gene annotations in hg38; the orange, red, and purple lines indicate two isoforms of RET, and the brown line is CCDC6. **b** CCDC6-RET with read alignments shown as grey lines. Further, some alignments do not support the inversion, suggesting that CCDC6-RET is heterozygous. **c** The entire picture of the inversion that caused CCDC6-RET. This inversion is too large to span by a single read; thus, it was identified as two independent fusion events at both the ends of the inversion, which would be difficult to understand if the two fusion events are visualized separately. The red line is a translocation that was not analyzed in this study. **d** Putative evolution process of LC-2/ad at the CCDC6-RET site. First, a long inversion generated two fusion genes, CCDC6-RET and RET-CCDC6. Second, a large deletion caused the loss of RET-CCDC6

The distance between RET (chr10: 43,075,069-43,132,349) and CCDC6 (chr10: 59,786,748-59,908,656) is about 17 Mbp in GRCh38. We confirmed that a CCDC6-RET fusion gene exists in LC-2/ad (Fig. 3A). This fusion gene is presumably caused by an inversion, although only one end of the inversion was found. We found an unknown novel adjacency that well explains the other end of the inversion (Fig. 3B, Additional file 7: Table S4). MoMI-G was able to display the relationships between the two breakends of the inversion, enabling users to understand large SVs. We explored the read alignments around the fusion and found that the fusion was heterozygous (Fig. 3C). MoMI-G is the first stand-alone genome graph browser that can display long-read alignments over branching sequences that represent a heterozygous SV.

Further, we found that the large inversion was flanked by two small deletions. These deletions are explained by a single deletion event following the large inversion event (Fig. 3D). The loss of the RET-CCDC6 fusion gene corresponds to the two small deletions on GRCh38. A simple explanation is that a deletion occurred after the inversion event, but not vice versa, in favor of the smaller number of mutation events.

Next, we attempted to estimate the breakpoints of the large inversion before the deletion occurred. There were two possible scenarios for the positions of the two breakends of the large inversion. The first is that RET-CCDC6 and CCDC6-RET were generated by a large inversion and then RET-CCDC6 was lost. The second is that CCDC6 was first broken by a large inversion, and a subsequent small deletion led to CCDC6-RET. Previous studies support the former scenario. First, the RET gene often tends to be disrupted in thyroid cancer by paracentric inversion of the long arm of chromosome 10, or by chromosomal fusion [32]. Second, in a previous study, two clinical samples had both RET-CCDC6 and CCDC6-RET in the genome [33]. Both studies suggested that an inversion disrupted both CCDC6 and RET, and then a small deletion disrupted RET-CCDC6. We could never recognize these two deletions flanking the large inversion without simultaneously observing both the inversion records in VCF.

### Nested SVs with alignment coverage

**Fig. 4:**
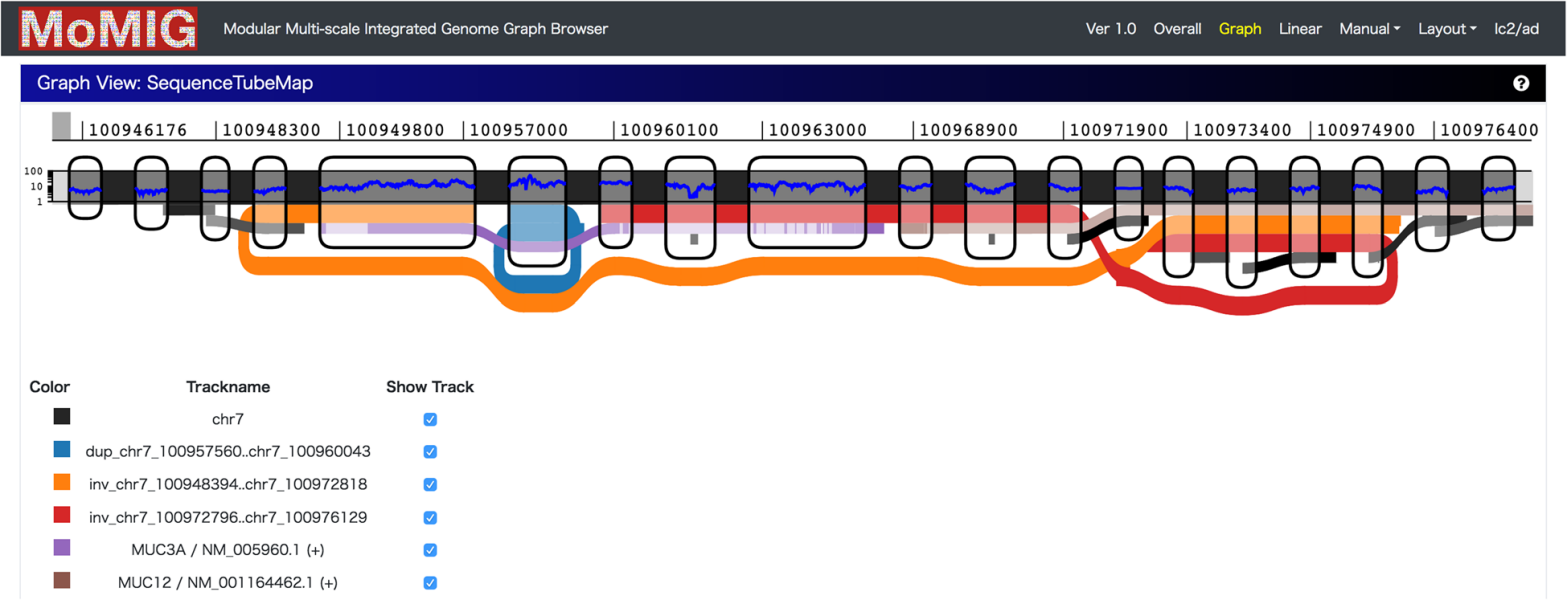
Nested SVs called by Sniffles in LC-2/ad. The thin black lines are repeat annotations. The brown and purple lines are gene annotations. The red and orange lines are an end of an inversion called by Sniffles. There are two possibilities for the genome structure: one is that MUC3A and its flanking region are a duplication, and the internal region of MUC12 is an inverted duplication. The other is that MUC3A and its flanking region are an inverted duplication, and the internal region of MUC12 is a duplication. Several read alignments support the former interpretation. Although SVs called from the Illumina reads did not include any of the SVs shown here, the alignment coverage by the Illumina reads is consistent with both duplications. Note that the y-axis of the blue thin line on the chromosome showing the alignment coverage is logarithmic.

Visualizing nested SVs is necessary for evaluating the output of SV callers. However, most existing genome browsers cannot visualize nested SVs as well as the relationships between them. Genome browsers, including ﻿Integrative Genomics Viewer (IGV [25]), collapse SVs into intervals between breakpoints, and thus the topological relationships between nested SVs are not shown. MoMI-G can visualize nested SVs as a variation graph (Fig. 4).

### Visualizing nested SVs in a pseudodiploid genome

**Fig. 5:**
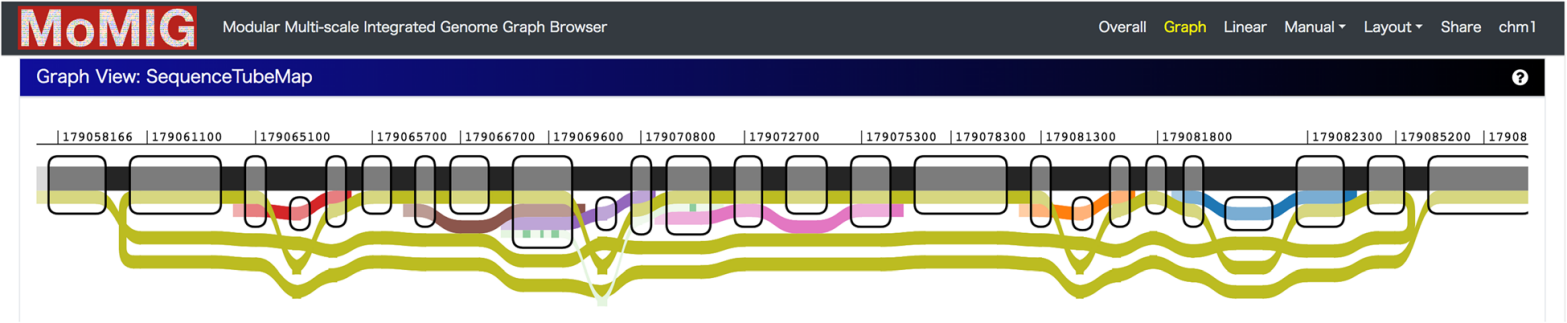
Nested SVs in CHM1. The black line represents a part of chromosome 5, where a large inversion is shown as the brown line. The other lines are smaller SVs included in the large inversion. Because CHM1 is a pseudodiploid genome, all the SVs shown in this figure must be on the same haplotype, although MoMI-G tools assume diploid (polyploid) genomes and show the inner SVs as heterozygous SVs

We show an example of nested SVs in a pseudodiploid genome visualized using MoMI-G. We downloaded a CHM1 genome with an SV list previously generated in a whole-genome resequencing study with PacBio sequencers from human hydatidiform [20]. The SV list includes insertions, deletions, and inversions for GRCh37/hg19. We converted the BED file of the SV list of CHM1 to a VCF file with custom scripts, and then filtered out deletions of less than 1000 bp to focus on medium to large SVs. We found nested SVs for which existing genome browsers do not intuitively show the relationships between them (Fig. 5). This example indicates that four insertions and deletions occur in the large inversion.

## User-interface design

The optimal way of visualizing SVs might vary. To rapidly explore the distribution of SVs in a genome, users might wish to use Circos-like plots. Other users might intend to focus on local graph structures of SVs that contain a few genes. In another scenario, a user might want to explore individual nucleotides. To address this issue, MoMI-G provides a customizable view in which users can place any combination of view modules. Further, preset view layouts are available for users’ convenience.

### Enabling easy manual inspection of detected SVs

Manual inspection, which includes determining if an SV is heterozygous or homozygous, confirming what part of a gene is affected by that SV, and determining the reason why an SV is called based on read alignments, is an important part of validating called SVs. As variants called by SV callers increase, the burden of manual inspection also increases, underscoring the importance of visualization both to inspect individual SV calls for filtering out false positives and to ensure that a filtered set of SVs is of high confidence [34, 35]. MoMI-G helps with the efficient inspection of SVs by applying (1) Feature Table, which is an SV list, (2) Interval Card Deck, which is genomic coordinate stacks, and (3) shortcut keys. The usage is as follows: (1) one can filter SVs using Feature Table, after which SVs are selected, and then (2) the listed variants are stacked on Interval Card Deck at the bottom of the window. In Interval Card Deck, intervals are displayed as cards, and the interval at the top (leftmost) card of the deck is shown on SequenceTubeMap. Each card can be dragged, and the order of cards can be changed. If one double-clicks on a card, the card moves to the top of the deck. A tag can be added for a card for later reference. Further, a card can be locked to avoid unintended modification or disposal, and the gene name can be input with autocompletion for specifying the interval of a card.

When the interval to view is changed, only part of the view that needs an update is re-rendered, whereas most genome browsers working on web interface require rendering the entire view. Interval Card Deck enables the rapid assessment of hundreds of intervals. Moreover, deciding whether an SV should be discarded or held becomes easier with shortcut keys. After all SVs are inspected, a set of SVs held on the Interval Card Deck is obtained, which might be a set of interesting SVs or a set of manually validated SVs. MoMI-G enables the rapid inspection of hundreds of SVs, providing a tool for validating hundreds of SVs or for selecting interesting SVs.

### Input requirements

MoMI-G inputs an XG format as a variation graph. Users can specify a GAM file with an index that contains read alignment. They can convert a BAM file into GAM using MoMI-G tools or can generate the GAM file on their own. When a BED or GFF file of genes is provided, users can specify a genomic interval by gene name. A configuration file is written in YAML. MoMI-G also accepts bigBed and bigWig formats [36] for visualizing annotations (e.g., repeats, genes, alignment depth, and GC content) on the reference genome. The bigBed and bigWig need to be extended for genome graphs in the future. The list of formats that are accepted by MoMI-G is shown in Table 1.

**Table 1:** MoMI-G data files

File type	Extension	Description
A succinct index of variation graphs	.xg	Variation graphs displayed in MoMI-G.
Graphical alignment/map	.gam	Read alignment. (optional)
Comma-separated values	.csv	SV list for chromosome-scale view. (optional)
Browser extensible data General feature format	.bed .gff	Used for converting gene names to genomic intervals. Also used for autocompletion of gene names. (optional)
Compressed binary indexed BED	.bb	Annotations. (optional)
Compressed binary indexed wiggle	.bw	Annotations. (optional)

## Discussion

We developed a genome graph browser, MoMI-G, that visualizes SVs on a variation graph in a way that researchers can assess the quality of SVs. Existing visualization tools for SVs show either one SV at a time, or all SVs together; the former does not allow the understanding of the relationships between SVs, whereas the latter is useless when the target genome is very large and the whole variation graphs are too complicated to view in a single screen (i.e., the hairball problem). MoMI-G allows viewing only part of the genome, which resolves the hairball problem, while providing an intuitive view for multiple SVs, including large and nested SVs. Further, MoMI-G enables the manual inspection of complex SVs by providing integrated multiple view modules; users can filter SVs, validate them with read alignments, and interpret them with genomic annotations.

We used vg as a server-side library and SequenceTubeMap as a client-side library for subgraph retrieval and visualization of genome graphs, because, to our knowledge, these are the only combinations that are publicly available. We found that significant amounts of engineering efforts are required for an even better user experience. For example, vg is a standalone command line application, not a daemon process; therefore, the current implementation of vg cannot keep the succinct index (this is actually the entire XG file) on memory in a similar way to daemon processes that persist over time. Every time a part of the genome graph is retrieved, the entire succinct index of several gigabytes is loaded. Once vg responds to a query, the loaded index is freed when the vg process exits. This is unnecessary overhead that will be eliminated when vg implements a daemon (server-client) mode, which we asked the authors of vg to implement. SequenceTubeMap displays inversions and duplications as loops; however, we found that new users occasionally find it difficult to recognize the connections between nodes. Visualizing SVs in an intuitive manner is still an open problem.

The currently available tools and formats for SV analysis have many problems. First, different SV callers output different VCF records even for the same SV. For example, depending on SV callers, an inversion with both boundaries identified at a base pair level is represented by one of the following: (1) a single inversion record, (2) two inversion records at both ends, (3) two breakend records at both ends, or (4) four breakend records at both ends (a variant of (3), but the records are duplicated for both the strands). Thus, developing a universal tool for variant graph construction is difficult. Second, certain types of nested SVs, such as an insertion within another insertion are impossible to represent in a VCF file, although variant graphs can easily handle these SVs. Imagine an Asian-specific sequence contains an Alu insertion of a low allele frequency. In the variant graph approach, this would be naturally represented as an Asian-specific sequence node with an Alu insertion of low frequency. In the current VCF format, this would result in insertion records of the two different sizes without the relationship between the two insertion sequences. Therefore, generating a variation graph from a VCF file including SVs is not ideal. We need an SV caller that directly outputs variation graphs.

Fostering the ecosystem around variation graphs is important for delivering their benefits to end users, as noted in the ecosystem around the SAM/BAM formats that spurred development of production-ready tools for end users. MoMI-G is the first step toward such a goal, because the availability of tools ranging from upstream analysis, such as read alignment, to visualization, is critical for the entire ecosystem.

The genome graph can have other future applications. For example, tools for visualizing isoforms or alternative splicing of genes, such as sashimi plots [37], can be extended to support “gene graphs”, where branches and merges are allowed in the gene model. Combining the idea of gene graphs and the database of variations in the human transcripts with the assessment of the impact of each variation on the 3D structures of proteins such as PhyreRisk [38] might also be interesting in the future. Another example might be showing assembly graphs. Tools such as Bandage [39], SGTK [40], and AGB [41] can show the ambiguity in genome assembly by visualizing assembly graphs, but read alignments on the graph are not shown. MoMI-G is potentially useful for showing assembly graphs with read alignments.

This is the one million genome era that requires rapid and memory-efficient data structure, since storing genomes naively consumes too much storage. To store the haplotype information of many individuals, shared sequences between individuals must be compressed; only variations between haplotypes must be stored. In this sense, the genome graph approach is considered promising, especially for human variation analysis. Indeed, recent studies showed more than one thousand of haplotypes can be stored on an index of the graph under certain assumptions [42, 43], although they still need improvements to fully capture the complex nature of the human genome diversity. We focused on SVs in this paper, but the current implementation of MoMI-G can visualize pangenomes if such data is available as GFA or XG files with paths. Considering efforts that catalog SVs from many individuals or samples at an unprecedented scale [44, 45], human pangenomes will be created in the near future, although how to construct a large human pangenome graph from a set of SVs between many individuals is still an open question. A full human pangenome with all variations including minor variations might be too complex to view without simplification. New methods for visualizing pangenomes for many individuals that give us the birds-eye view should be developed. MoMI-G is a step forward for visualizing genome graphs and allows the development of new analysis algorithms on genome graphs.

## Conclusions

We developed a genome graph browser, MoMI-G, which is the first-in-class genome browser that allows users to view a human-sized genome graph both with branches and with annotations/read alignments. This enables us to visualize complex and large structural variations identified by long-read sequencing and analysis more smoothly and more intuitively. In addition, users can easily filter out false positives by manually inspecting hundreds of identified structural variants with supporting long-read alignments and annotations in a short time.

## Methods

### Datasets

The LC-2/ad cell line was obtained from RIKEN BRC, and was cultured as previously described in [17]. HMW gDNA was extracted from lung cancer cell line, LC-2/ad by using a Smart DNA prep (a) kit (Analykjena). Whole-genome shotgun data were produced from MinION 1D sequencing (SQK-LSK108), MinION 1D^2 sequencing (SQK-LSK308), and MinION Rapid sequencing (SQK-RAD003). For MinION 1D sequencing, 4 μg HMW gDNA was quantified using Tape Station. DNA repair was performed using NEBNext FFPE DNA Repair Mix (M6630, NEB). End-prep was performed using NEBNext Ultra II End Repair/dA-Tailing Module (E7546L, NEB). Adapter ligation was performed using NEBNext Blunt/TA Ligase Master Mix (M0367 L, NEB) and the Ligation Sequencing Kit 1D (SQK-LSK108, Oxford Nanopore Technologies). Libraries were sequenced for 48 h with MinION (R9.5 chemistry, Oxford Nanopore Technologies). For MinION 1D^2 sequencing, the protocol was the same as that for 1D excluding adapter ligation by using Ligation Sequencing Kit 1D^2 (SQK-LSK308, Oxford Nanopore Technologies). The library for MinION Rapid sequencing was prepared according to Sequencing Kit Rapid (SQK-RAD003, Oxford Nanopore Technologies).

### Nanopore data alignment

Nanopore WGS data were aligned against the GRCh38 human reference genome by using NGM-LR with “-x ont” option (version 0.2.6) for calling SVs with Sniffles version 1.0.7 [5]. MinION 1D^2 sequencing can produce a 1D^2 read, which integrates the information of a read and its complementary read into one read. MinION 1D^2 sequencing produces two types of fastq files, 1D and 1D^2; there was some redundancy between 1D and 1D^2 reads. Therefore, redundant 1D reads were removed, and only 1D^2 reads were used. Moreover, some redundant 1D^2 reads were found. These reads were removed from 1D^2 files and used as 1D reads. The percentage of the primary aligned reads was 54.7% (Additional file 5: Table S2). This is because we did not filter out reads excluding 1D-fail reads of Rapid sequencing for alignment.

### SV calling

Sniffles (version 1.0.7) with a parameter “-s 5” was used to call SVs. The minimum number of supporting reads was determined such that we could detect the known large deletion of CDKN2A [17], but alignment bias for a reference genome reduces the call rate of insertions compared to that of deletions [2, 20]. We detected 11,316 records as a VCF file of SVs, including CCDC6-RET, on the nuclear DNA of LC-2/ad cell line (Additional file 6: Table S3). Illumina HiSeq 2000 Paired-end WGS data (DDBJ accession number, DRX015205) aligned against GRCh38 by using bwa (version 7.15) [46] were used for calling SVs with Lumpy (version 0.2.13) [47], delly (version 0.7.7) [48] and manta (version 1.0.3) [49]. The results of these SV callers are listed in Additional file 8: Table S5. The four lists of SV candidates detected using Sniffles, Lumpy, delly, and manta were merged using SURVIVOR (version 1.0.0) [50] with “5 sv_lists 1000 1 1 0 0” option for clustering SV candidates (Additional file 9: Figure S4). Further, we filtered the merged candidates based on the following three criteria: (1) Remove SVs of 0 bp to 1 kbp in length to focus on large SVs, (2) Remove all insertions and non-canonical SV types: INVDUP, DEL/INV, and DUP/INS (technically unnecessary, but we aimed at cross-validating SV candidates from Illumina reads with which insertions and non-canonical SV types are hard to call), and (3) Remove SVs overlapping with the intervals of the 10X default blacklist (https://github.com/10XGenomics/supernova/blob/master/tenkit/lib/python/tenkit/sv_data/10X_GRCh38_no_alt_decoy/default_sv_blacklist.bed) for reducing false positives. After filtering, we obtained 1790 SV records.

### Constructing a variation graph

We constructed a variation graph, including a reference genome (GRCh38) and an individual genome (either LC-2/ad or CHM1). We developed scripts that we call MoMI-G tools. MoMI-G tools follow the procedure in SplitThreader Graph [51].

1. Extract a breakpoint list from a VCF file generated by Sniffles.
2. Construct an initial variation graph with each chromosome of the GRCh38 primary assembly as a single node.
3. Split nodes every 1 Mbp due to the limitation in the implementation of vg used during the development of MoMI-G; otherwise, vg aborted with an error. The latest version of vg does not have this limitation.
4. For each breakpoint in the breakpoint list, split the node of the graph that contains the breakpoint into two nodes at the breakpoint.
5. Create reference paths that represent the chromosomes in the reference genome.
6. Create SV paths that represent SVs, each of which corresponds to one record in the input VCF file. Further, add a node to the graph when the type of SV is an insertion.

Because there was no publicly available tool that converts VCF files produced by existing SV callers into GFA format, we wrote custom scripts, MoMI-G tools, for converting the SV output by Sniffles into the input file of MoMI-G. At the moment, the MoMI-G tools support the output of Sniffles, LongRanger, and SURVIVOR. SURVIVOR can merge and compare the output of multiple SV callers such as pbsv; the MoMI-G tools indirectly supports such SV callers. Other tools may work if the output is compatible with one of the supported tools, but we did not test it. We tried supporting other SV callers, but how each SV caller encodes an SV into a VCF record may vary and is undocumented, which we call “VCF dialect issue.” The representation of SVs varies between SV callers: the same SV can be described in different ways in the current VCF format. For example, Sniffles outputs an INV record to indicate only one end of the inversion, but some other tools uses an INV record to indicate a single inversion (both ends included). Other tools output a BND record to indicate a breakend, only one end of an inversion. The variety of the interpretation makes it necessary for us to do additional extensive tests for each SV callers to support other SV callers. Other graph construction tools for SVs suffer from the same issue. For example, vg has a “construct” subcommand that constructs a variation graph from a pair of a reference genome and a VCF file, but “vg construct” was incompatible with the output of all SV callers we know of for the reason stated above, at least when we were developing the MoMI-G tools.

In general, to display read alignments, we recommend aligning against a variation graph by directly using “vg map” instead of converting read alignments against the linear reference genomes into the GAM format because it gives better alignment results. Nevertheless, we intended to inspect the results of Sniffles; therefore, we wrote a custom script to convert alignments against the linear reference (BAM file) into a GAM file by using “vg annotate.” Although MoMI-G can display the individual nucleotides of aligned reads (users can see single nucleotide variants in individual reads) when CIGAR strings are stored in the input GAM file, such examples are not shown in this paper. GAM files converted from BAM files by the MoMI-G tools do not contain the CIGAR string due to the limitation of “vg annotate”; “vg annotate” was originally designed for placing annotations in BED/GFF files on paths on variation graphs and not for alignments. We hope that the MoMI-G tools are soon replaced by future genome-graph-aware SV callers that outputs a genome-graph-aware variant format that is completely free from the VCF dialect issue. One possible solution to this issue is making SV callers graph-aware, which we hope to see in the near future.

### Inspection of SV candidates

We modified and integrated SequenceTubeMap into MoMI-G so that it can visualize a variation graph converted from SVs that shows the difference between a reference genome and an individual genome. The modifications made to SequenceTubeMap are shown in Additional file 10: Table S6. One can click on the download button for downloading an SVG image generated by SequenceTubeMap so that a vector image can be used for publication.

### Visualizing annotations

Ideally, MoMI-G provides annotations on variation graphs. However, annotations available in public databases are for the linear reference genome. MoMI-G can display annotations in the bigWig/bigBed formats. In particular, for human reference genomes, GRCh19/hg37 and GRCh38/hg38, MoMI-G provides an interface for retrieving Ensembl gene annotations from the Ensembl SPARQL endpoint [52] via SPARQList REST API (https://github.com/dbcls/sparqlist). The orientation of genes is shown in the legend of SequenceTubeMap. Further, if one clicks on a gene name, the website of the gene information in TogoGenome [53] opens.

### Miscellaneous modules

Threshold Filter: Threshold Filter has two use cases: First, one can toggle checkboxes to select whether to show inter-chromosomal SVs and/or intra-chromosomal SVs; second, one can filter SVs with a slider based on the custom priority (possibly given by SV callers) of each SV.

Annotation Table: Annotation Table shows all annotations that are displayed on the SequenceTubeMap. Moreover, annotations can be downloaded as a BED file.

Linear Genome Browser: To provide a compatible view of a selected genomic region, we integrated Pileup.js [54] into MoMI-G.

### Backend server

The MoMI-G backend server caches subgraphs once a client requests a genomic interval on a reference path to avoid querying the same query many times for vg backend. The MoMI-G server retrieves the subgraph from the index every time users change to a new (not cached) region. To mitigate this issue, the MoMI-G server caches subgraphs when a client requests a genomic interval on a path. This caching mechanism helps us avoid wasting time in redundant work by vg; cached region can be rendered quickly without spawning the heavy backend vg process that consumes a long time for loading the huge index. The MoMI-G server then retrieves annotations from bigWig and bigBed with a range query and provides JSON API with which the client can make queries.

## Availability and requirements

Project name: MoMI-G.

Project home page: https://github.com/MoMI-G/MoMI-G

Operating systems: GNU/Linux (server), platform-independent (web application; client).

Programing language: Rust (server), TypeScript (client).

Other requirements: Web browser with support for ECMAScript 5 (client).

License: MIT.

Any restrictions to use by non-academics: None.

## Supplementary information

**Additional file 1: Figure S1.** A representative screenshot of MoMI-G with all view modules.

**Additional file 2: Figure S2.** An example of base-to-base alignment information on MoMI-G.

**Additional file 3: Table S1.** List of MoMI-G features.

**Additional file 4: Figure S3.** Examples of a deletion, balanced inversion, and duplication. (A) Deletion: The orange line indicating a deletion starts before one breakend of the deletion, passes through the middle node that indicates the deleted sequence, and then proceeds for the sequence flanking the deletion. (B) Balanced Inversion: The purple line indicating a balanced inversion includes flanking sequences on both breakpoints of the inversion. (C) Duplication: The blue line indicating a duplication passes through the node twice, suggesting that the sequence of the node is duplicated. The line in the node might terminate if the node is interrupted by other SVs.

**Additional file 5: Table S2.** Summary of the Oxford Nanopore sequencing data of LC-2/ad.

**Additional file 6: Table S3.** Called SVs in LC-2/ad nuclear DNA from Nanopore reads.

**Additional file 7: Table S4.** Breakends in the opposite end of the CCDC6-RET inversion.

**Additional file 8: Table S5.** Called SVs in LC-2/ad nuclear DNA from Illumina reads.

**Additional file 9: Figure S4.** Merged SVs from four SV callers by using SURVIVOR. The green circle describes the count of SVs called by Illumina, whereas the red circle presents the count called by MinION.

**Additional file 10: Table S6.** The modifications made to the original SequenceTubeMap.

## Data Availability

Newly obtained long-read sequencing data of LC-2/ad were deposited in the DDBJ with accession numbers DRA007941 (DRX156303-DRX156310). Datasets included in this article are also provided in the database, DBTSS/DBKERO [55]. The source code of MoMI-G is available at https://github.com/MoMI-G/MoMI-G/ under the MIT license. It is written in TypeScript and was tested on Linux and Mac operating systems. Further, we have included an example dataset and annotations as a Docker image.

## Abbreviations

API: Application programming interface
BAM: Binary alignment map
BED: Browser extensible data
BND: Breakend
DEL: Deletion
DUP: Duplication
GAM: Graph alignment map
GFF: General feature format
HMW: High-molecular-weight
INS: Insertion
INV: Inversion
REST: Representational state transfer
SAM: Sequence alignment map
SV: Structural variants
VCF: Variant call format
