# Supplementary figures and images for: MoMI-G: modular multi-scale integrated genome graph browser
Source: BMC Bioinformatics. 2019 Nov 5;20:548. doi: 10.1186/s12859-019-3145-2 (PMC6833150; doi:10.1186/s12859-019-3145-2)

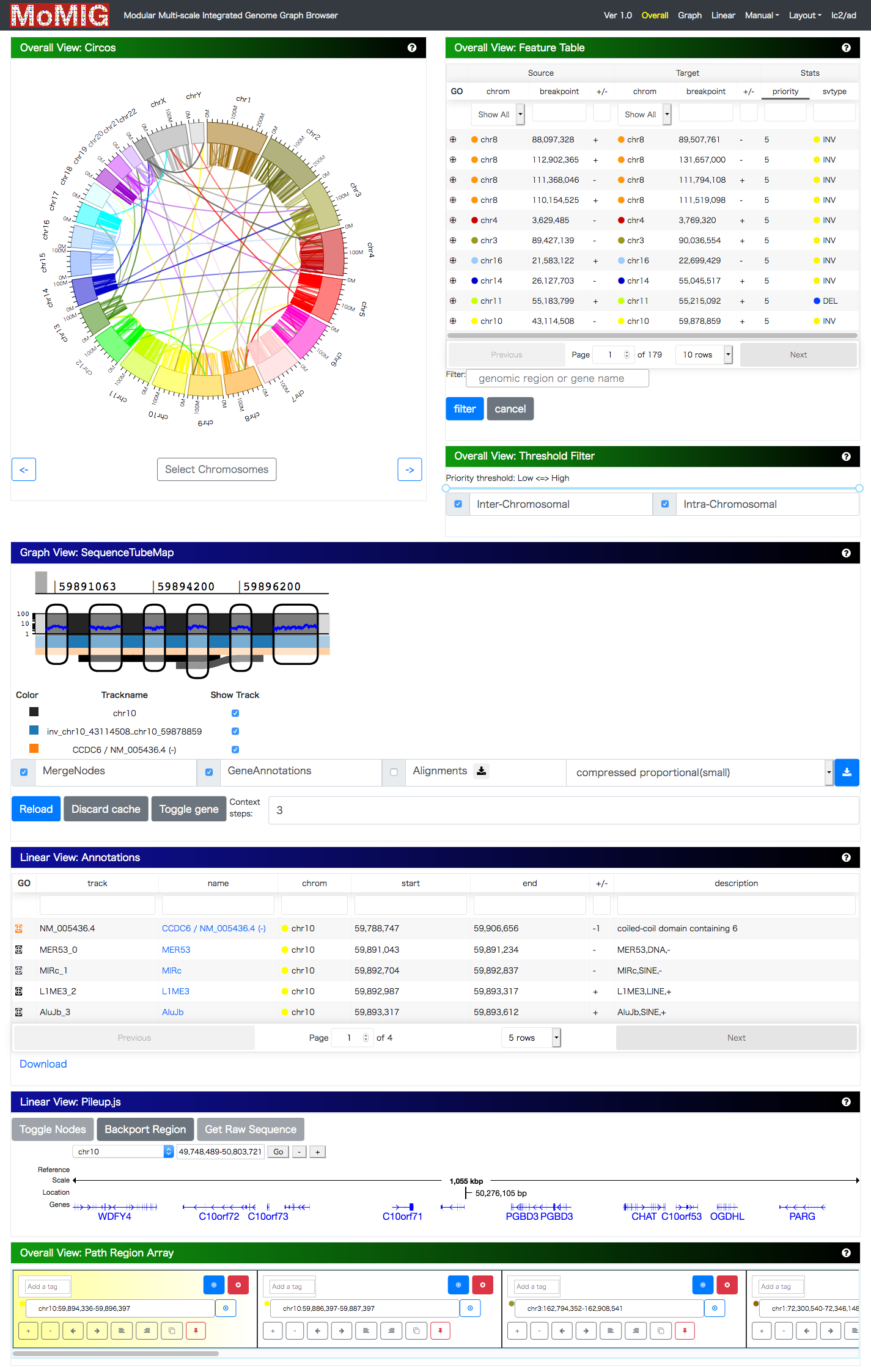

Supplement: Supplementary file 1 — Additional file 1: Figure S1. A representative screenshot of MoMI-G with all view modules. [file 12859_2019_3145_MOESM1_ESM.png]

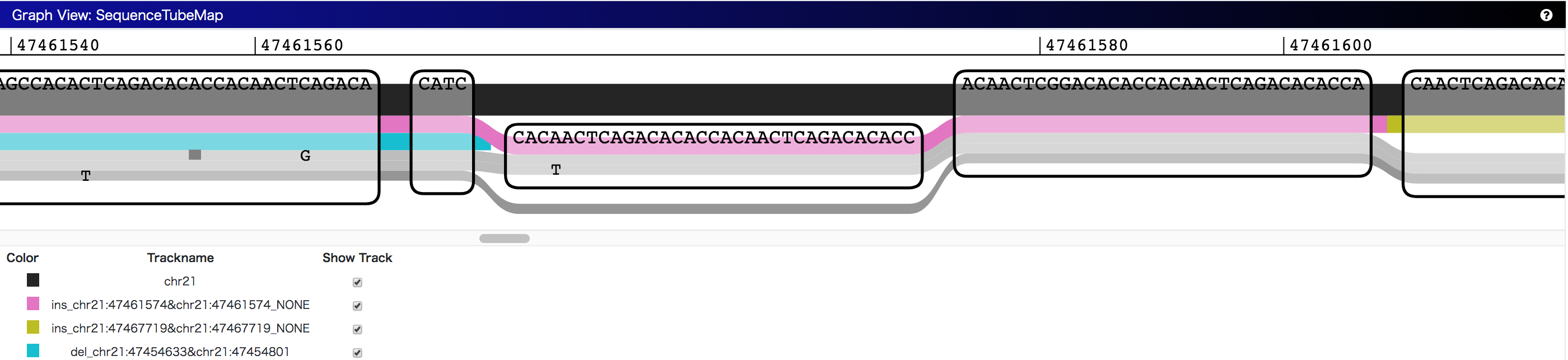

Supplement: Supplementary file 2 — Additional file 2: Figure S2. An example of base-to-base alignment information on MoMI-G. [file 12859_2019_3145_MOESM2_ESM.png]

A

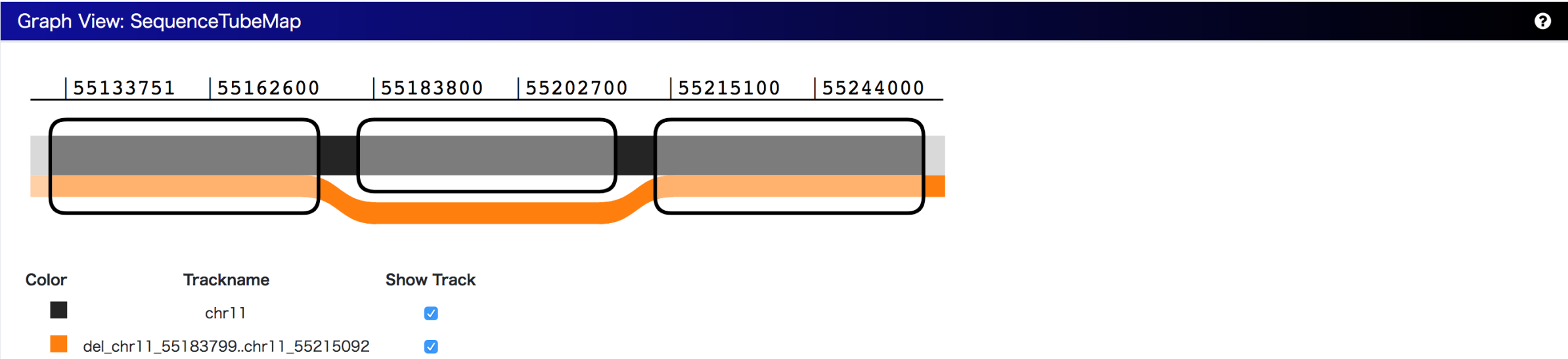

B

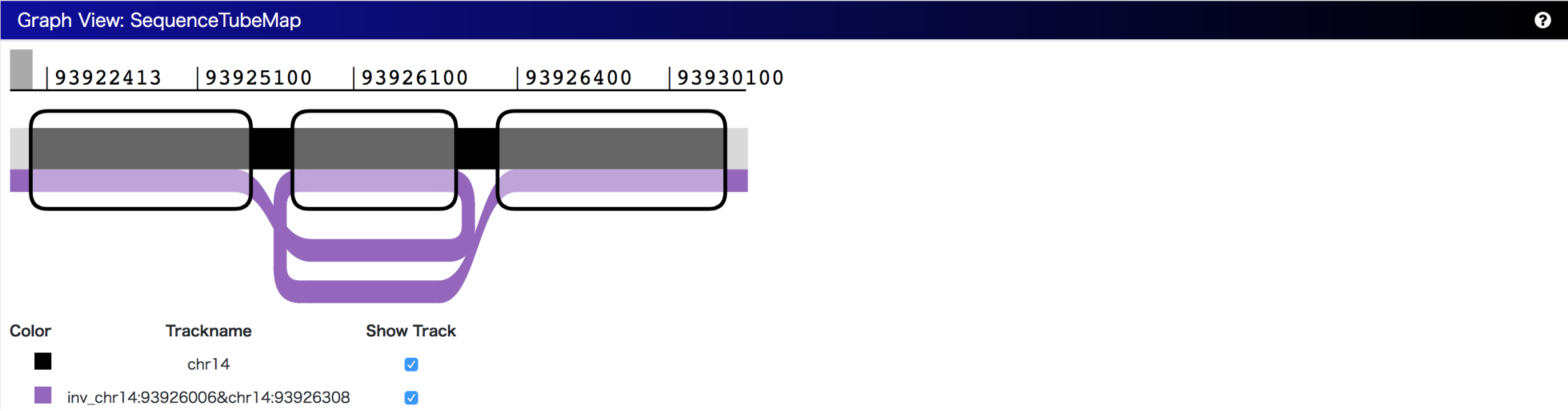

C

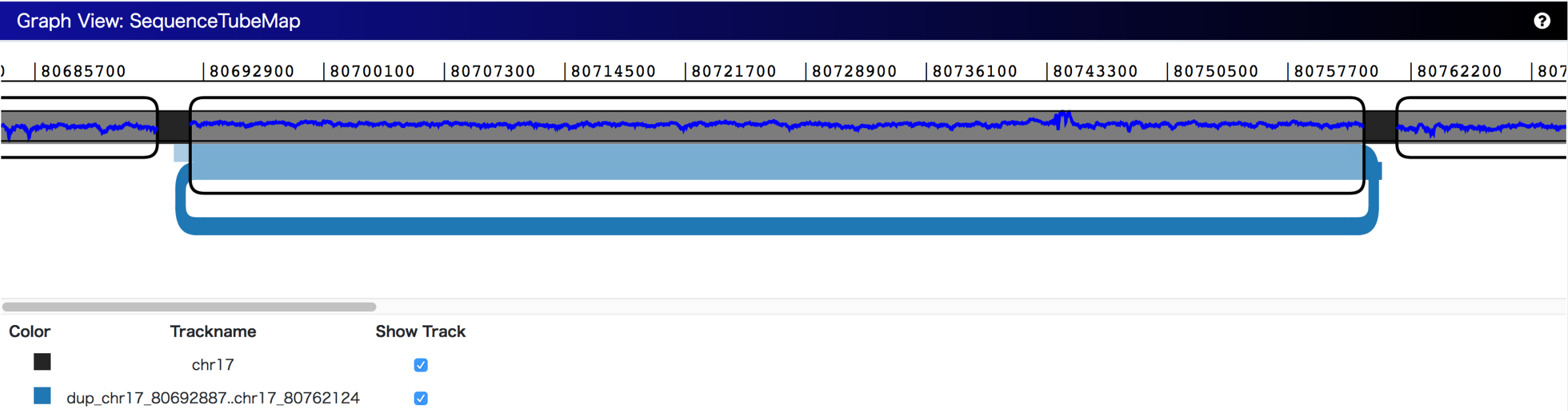

Supplement: Supplementary file 4 — Additional file 4: Figure S3. Examples of a deletion, balanced inversion, and duplication. (A) Deletion: The orange line indicating a deletion starts before one breakend of the deletion, passes through the middle node that indicates the deleted sequence, and then proceeds for the sequence flanking the deletion. (B) Balanced Inversion: The purple line indicating a balanced inversion includes flanking sequences on both breakpoints of the inversion. (C) Duplication: The blue line indicating a duplication passes through the node twice, suggesting that the sequence of the node is duplicated. The line in the node might terminate if the node is interrupted by other SVs. [file 12859_2019_3145_MOESM4_ESM.pdf]

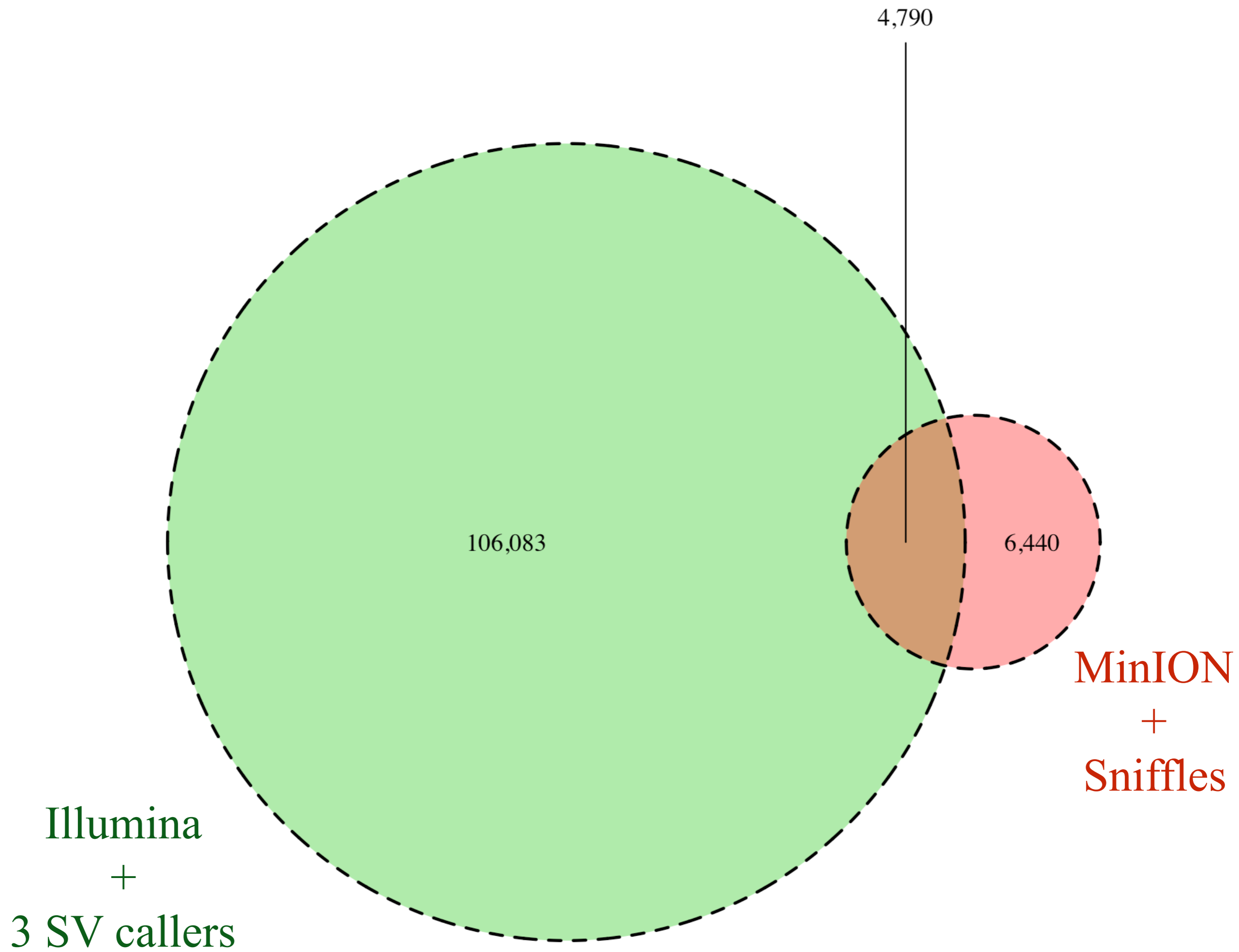

Supplement: Supplementary file 9 — Additional file 9: Figure S4. Merged SVs from four SV callers by using SURVIVOR. The green circle describes the count of SVs called by Illumina, whereas the red circle presents the count called by MinION. [file 12859_2019_3145_MOESM9_ESM.pdf]
